# De La Leucocythémie Chez Les Animaux Domestique

**Published:** 1881-04

**Authors:** 


De la Leucocythémie chez les Animaux Domestique. Par M. Ed. Nocard, Professeur de Clinique a l'Ecole Veterinaire d'Alfort. Paris: Asselin & Cie. 1881.

*Leucocythaemia in the Domestic Animals.*—In this monograph by Dr. Nocard, we have presented a very complete *resumé* of the whole subject of leucocythæmia. The author reviews the history and pathology of the disease, and then gives an abstract of all the cases which have been reported as occurring in the lower animals. This summary, which is extremely interesting, shows a prevalence of the disease among these animals that is not generally known. He reports nine cases as occurring among horses, six among cows, five among swine, twenty-one among dogs, and one in a cat. The morbid anatomy of the disease in these animals is described. We notice no striking difference, however, between lesions here and those in the human body. The increase of white globules in the blood is in about the same ratio. The spleen and the lymphatic glands and other organs are affected also in the same way. The most frequent lesion is hypertrophy of the spleen, next come the lymphatic glands, and then the liver. The symptoms of the disease are weakness and loss of appetite, sometimes evidences of pain in the left hypochondrium, dyspnœa, hemorrhage from the nose or intestines, loss of flesh, in some cases ascites, and occasionally polyuria. In splenic and in lymphatic leucocythæmia the evidences of the disease are shown by the enlarged glands. Death inevitably results.

The author states that inoculation of the lymphatic products in living animals has been tried without result. This, he says, is one test of its entire distinctness from tuberculosis, with which disease certain authors appear to have confounded it, especially when it affected the lungs.

With reference to treatment, he recommends no specific measure. Iron and tonics and good food will produce some relief. Splenotomy and extirpation of the enlarged lymphatic glands does no good.

